# Novel Therapeutic Targets in Axial Spondyloarthritis

**DOI:** 10.1007/s40674-018-0095-1

**Published:** 2018-04-12

**Authors:** Claudia Worth, Paul Bowness, M. Hussein Al-Mossawi

**Affiliations:** Nuffield Department of Orthopaedics Rheumatology and Musculoskeletal Sciences, Botnar Research Centre, University of Oxford, Oxford, OX3 7HE UK

**Keywords:** Spondyloarthritis, Th17, GM-CSF, JAK, ROR-γt

## Abstract

**Purpose of review:**

Axial spondyloarthritis remains an area of significant unmet clinical need with only two immune pathways currently targeted by licenced therapies compared to other immune-mediated inflammatory joint disorders such as rheumatoid arthritis where a multitude of therapeutic options are available. This review will look at emerging therapeutic targets in axial spondyloarthritis beyond the neutralisation of IL-17A and TNF by monoclonal antibodies.

**Recent findings:**

Several promising targets are in various stages of pre-clinical and clinical development in axial spondyloarthritis. These include small molecule approaches to target transcription factors, epigenetic modification and intracellular modulation of cytokine signalling by kinase inhibition. GM-CSF has also emerged as a potential driver of inflammation.

**Summary:**

A number of novel and promising therapeutic options are in various stages of development in axial spondyloarthritis. The Janus kinase inhibitors have shown great promise in other immune-mediated inflammatory disorders and will be an exciting addition to the axial spondyloarthritis field as the first oral disease-modifying agents. GM-CSF blockade also shows great promise since antibodies for neutralising this cytokine are safe in patients and have shown efficacy in other immune-mediated inflammatory diseases.

## Introduction

Axial spondyloarthritis (SpA) is an inflammatory disease affecting up to 0.5% of the population in North America and Europe and encompasses ankylosing spondylitis (AS) [1, 2]. The disease has a strong association with HLA-B27 inheritance [3] and typically causes inflammation in the spine and sacroiliac joints [4]. This inflammation can progress to new bone formation and eventual fusion of the spine in severe cases [5]. Despite recent advances, treatment options in this disease remain limited compared to rheumatoid arthritis. Physiotherapy and non-steroidal anti-inflammatory drugs (NSAID) are first-line therapies followed by biological anti-TNF, and more recently, anti-IL-17A therapies [6]. Currently, there are no approved therapies on the market for patients who are refractory to these two cytokine targets and thus a great unmet clinical need remains. There are several novel therapeutic approaches in development and this review will highlight some of these approaches beyond anti-IL-17A and anti-TNF. We will concentrate first on novel approaches to targeting “type 17 responses” (also referred to as type 3 immune responses), with a particular focus on the potential role of GM-CSF as a therapeutic target, before describing other approaches such as phosphodiesterase 4 inhibition.

## Targeting ROR-γt, a key type 17 transcription factor

The role of the IL-23/IL-17A axis in SpA pathogenesis was first suggested following genome-wide association studies implicating polymorphisms of the IL-23 receptor [7], and has now been proven by the success of anti-IL-17A therapies in the treatment of in AS [8•]. Thus far, focus on this axis has predominantly been on the role of IL-17A secreted by CD4 T cells, but it is now appreciated that this axis involves numerous additional effector cytokines. In particular, Th17 cells have been shown to co-produce IL-17F, IL-22, IFNγ, IL-26 and GM-CSF [9, 10]. Additionally, the axis is not only limited to the CD4 compartment and other cell types including CD8 [11], mucosal-associated invariant T (MAIT) cells [12], γδ T cells [13] and innate-lymphoid cells [14] which are all capable of activating this inflammatory programme. All these cell types share expression of the retinoid orphan-related receptor-γt (ROR-γt) and thus targeting this transcription factor would be an attractive approach for switching off this inflammatory module. The first reports of ROR-γt inhibitors used digoxin to inhibit this transcription factor in murine cells [15] but the doses required to achieve this inhibition in human cells would be toxic. Over the years, a number of small molecule ROR-γt inhibitors have been developed and tested in several in vitro Th17-driven systems. One such report showed ROR-γt inhibitors to be capable of supressing Th17 signatures from psoriatic patient samples [16], whilst another report showed inhibition of both IL-17A and IL-17F by another ROR-γt inhibitor (SR2211) [17]. We developed an assay to expand Th17 cells from the peripheral blood and synovial fluid of SpA patients and showed a clear inhibition of IL-17A by two ROR-γt inhibitors (MRL-367 and MRL-248) [18••]. In addition to the inhibition of secreted IL-17A, we also demonstrated a downregulation of a number of inflammatory genes associated with the Th17 programme including IL-17F, IL-22, and IL-26. Despite the early promise of a number of ROR-γt inhibitors, recently, reports of thymic aberrations in Lewis rats treated with ROR-γt inhibitor compounds [19] have raised fears over long-term safety. It remains to be seen whether this safety concern is a class effect or specific to one compound, and at the time of writing, there is currently one registered trial on clinicaltrials.gov for a ROR-γt inhibitor ointment actively recruiting but no trials of systemic administration of ROR-γt inhibitors. If ROR-γt inhibition does not prove to be safe, there are likely other molecular targets in or on Th17 cells that are worthy of investigation in the context of SpA.

## Epigenetic modulation of type 17 immune responses

Epigenetic modulation of key genes involved in Th17 differentiation and effector function, for example through the therapeutic targeting of bromodomains, is another area where novel therapies are currently being developed. Bromodomains recognise acetylated lysine residues in histones and thus play a very important role in the control of gene expression [20]. Pan-BET bromodomain inhibitors such as JQ1 have shown efficacy in Th17-driven murine models of inflammation including experimental autoimmune encephalomyelitis [21], but concerns remain about the specificity of such broad bromodomain inhibitors in clinical trials. In a study published by our group in collaboration with the Structural Genomics Consortium, we used a potent and very selective inhibitor for the bromodomains of the transcriptional coactivators CBP and its close relative p300 to inhibit Th17 responses from the peripheral blood of SpA patients [22•]. This approach was more selective compared to the pan-bromodomain inhibitor JQ1 and was successful in inhibiting IL-17A and GM-CSF but not IL-17F and IL-22. It is not clear if the sparing of IL-17F and IL-22 would be sufficient for switching off inflammation in an in vivo model, and the selective inhibitor CBP30 in its current form does not have the appropriate pharmacodynamics properties for such in vivo studies. Nevertheless, if safe and stable inhibitors could be delivered to inflammatory sites, this would be a promising therapeutic strategy.

## JAK inhibition

The Janus kinase (JAK) system of signalling downstream of cytokine binding has over recent years been an area of great interest in the treatment of inflammatory disorders. Recent positive results seen in rheumatoid arthritis for tofacitinib, a JAK1/3 (plus some JAK2 activity) inhibitor [23], and baricitinib, a JAK1/2 inhibitor [24], have for the first time offered the promise of biologic-like efficacy with an oral small molecule inhibitor. In spondyloarthritis, we have tested the effects of tofacitinib on the Th17 responses of patients with SpA using patient-derived PBMCs in vitro and have showed a significant inhibition in the amount of secreted IL-17A, IL-17F, IL-22, and GM-CSF [25]. These results are in agreement with the results of the recently published, placebo-controlled trial in ankylosing spondylitis where tofacitinib met its primary ASAS20 endpoint compared to placebo in a phase II 16-week dose-ranging study [26••]. However, the response was only significant for the 5mg twice daily group (80.8% response), while in the 2mg and 10mg twice daily groups the response was not significant at 51.9% and 55.8% respectively, compared to a placebo response of 40.1%. Moreover, the data also went against expectations for those achieving the higher bar of an ASAS40 response with 42.3% of those on the lowest 2mg dose achieving an ASAS40 response versus 38.5% of those on the highest 10mg dose. Crucially, the Pfizer pipeline currently (Pfizer.com/pipeline) does not show plans for a phase III study for tofacitinib in SpA and there are no active trials registered on clinicaltrials.gov for either tofacitinib or baricitinib. We think that this is a promising area for future research, although further studies are needed to determine which key members of the JAK/STAT family are involved in SpA pathogenesis, and whether selective or broad targeting of these is most appropriate.

## GM-CSF as a novel target in spondyloarthritis

GM-CSF is a haematopoietic growth factor and multifunctional cytokine [27]. Within the CD4 T cell compartment, GM-CSF production in the context of a Th17 response has been shown to be pathogenic in murine immune-mediated inflammatory responses [28, 29]. A more recent study showed neutralisation of GM-CSF can ameliorate inflammatory arthritis and lung inflammation in the SKG mouse model of SpA [30]. These immuno-modulatory properties of GM-CSF make it a novel therapeutic target in diseases characterised by immune dysregulation, such as RA and SpA.

GM-CSF gene expression can be induced by interleukin IL-1β, IL-6, TNF and endotoxin in a variety of cell types, including fibroblasts, endothelial cells, T cells, macrophages and epithelial cells [31]. GM-CSF exerts its effector function by binding to its cell surface receptor (GM-CSFR) which is coupled with JAK2, and the downstream mitogen-activated protein kinase (MAPK) and phosphoinositide 3-kinase (PI3K) signalling pathways [32, 33]. GM-CSFRs are predominantly expressed on myeloid cells but can also be found on some non-haematopoietic cells like fibroblasts [31].

In health, GM-CSF plays a critical role in haematopoiesis and in the immune response to injury and infection. GM-CSF shapes the interface between innate and adaptive immunity, by interacting with autocrine and paracrine cytokine networks [34] and by promoting myeloid cell survival, proliferation, activation and differentiation [35]. Furthermore, GM-CSF modulates subsequent adaptive immune responses by influencing antigen-presenting cell (APC) [36, 37] and T helper (Th) cell differentiation [38–41].

GM-CSF and its receptor have been shown to be overexpressed in synovial joints of patients with immune-mediated inflammatory arthritides like RA [36, 42] and SpA [43]. GM-CSF is thought to promote joint damage by recruiting granulocyte and macrophage precursors from activated bone marrow adjacent to synovial joints [44]. It then induces their differentiation into a more inflammatory phenotype and also activates monocyte-derived dendritic cell maturation [45]. In addition, GM-CSF causes the release of pro-inflammatory chemokines such as CCL17 [46]. GM-CSF also facilitates cartilage destruction and bone resorption by the induction of matrix metalloproteinases [47] and the osteoclast activating factor RANKL [48, 49]. Moreover, GM-CSF has been shown to be responsible for arthritic pain in mice independently of its pro-inflammatory characteristics and GM-CSF receptors have been shown to be expressed on nerve endings [50, 51].

Neutralising anti-GM-CSF monoclonal antibodies have been very effective in alleviating pain and in improving joint damage and inflammation in arthritic mice [30, 42]. This effect can be potentiated by concomitant blockade of IL-17A [52]. Furthermore, the benefits of GM-CSF blockade appear to be independent of IL-6 and TNF [30, 37, 53]. Five human monoclonal antibodies (mAbs) targeting the GM-CSF pathway are currently in phase I and II clinical trials in RA. Mavrilimumab, a high-affinity IgG4 monoclonal antibody (mAb) against the α-chain of GM-CSFR, has been evaluated in four phase II clinical trials [54, 55]. These comprise a total of 748 patients with moderate RA and an inadequate response to at least one disease-modifying anti-rheumatic drug (DMARD), usually methotrexate. Patients who failed one anti-TNF agent were also included. The ACR20 response improved significantly in a dose-dependent manner with mavrilimumab treatment (*p* < 0.001 when compared to placebo) [54]. Biomarkers in addition to pain and physical function scores also improved significantly [56]. The EARTH explorer II trial directly compared mavrilimumab with the anti-TNF agent golimumab in incomplete responders to DMARDs and/or one anti-TNF mAb. Whilst this study was not sufficiently powered to reach statistical significance, its findings nevertheless suggest that mavrilimumab is at least as effective as golimumab in these patients [57]. Moreover, benefits were maintained and no significant safety concerns were identified at 5-year follow-up in an open-label extension study comprising 442 patients [56]. Of note, phase I and II trials using mAbs that target GM-CSF directly, namely MOR103, namilumab and MORAb-022, are similarly encouraging [58]. Anti-GM-CSF/CSFR mAbs may therefore be a valuable novel biological treatment option; both as first-line therapy in anti-TNF naïve patients and as second-line therapy in anti-TNF non-responders. In addition, GM-CSF blockade may have significant analgesic benefits which would address a significant unmet need in inflammatory disorders. GM-CSF may be a very effective target in SpA based on the pre-clinical data from our group and others which show an expansion of GM-CSF-producing CD4 and CD8 cells in the blood and synovial fluid of patients with axial SpA [59••] compared to healthy donors and RA inflammatory control patients suggesting this pathway may be more relevant in SpA than RA.

## Other therapeutic targets

### Phosphodiesterase inhibition

One recent oral drug modulating inflammatory pathways is apremilast, a phosphodiesterase 4 (PDE4) inhibitor. PDE4 is the main class of phosphodiesterase expressed in T cells and inhibition of this enzyme leads to accumulation of cyclic AMP (cAMP). The persistence of cAMP in T cells after activation has been shown to decrease the amount of secreted IFNγ in vitro [60]. However, the role of cAMP in Th17 biology is less clear. Boniface and colleagues showed that cAMP was required for the differentiation of Th17 cells [61] in vitro and can be mimicked by the addition of an intracellular cAMP analogue [61], whilst the addition of apremilast to in-vitro T cell cultures broadly activated with anti-CD3 only was reported to decrease the amount of secreted IL-17A in the culture supernatants [62]. The clinical efficacy of apremilast has been somewhat mixed. In psoriasis [63] and psoriatic arthritis [64], apremilast has been shown to be effective in randomised placebo-controlled trials although the benefit in psoriatic arthritis seemed modest compared to biologic therapies with an ACR20 response rate of 38.1%. In ankylosing spondylitis, apremilast failed to meet its primary endpoint in a small phase II study but was associated with a numerically greater improvement from baseline for all clinical assessments [65]. A larger phase III study was undertaken, and results reported on clinicaltrial.gov show no difference in the ASAS20 primary outcome compared to placebo at 24 weeks. The failure of apremilast in ankylosing spondylitis may be due to patient heterogeneity or to the complexity of the underlying T cell biology and the exact role elevated cAMP levels play in Th17 differentiation and function.

### ERAP1 or HLA-B27 inhibition?

A number of other potential therapeutic targets have been suggested by genetic studies of AS including genome-wide association studies. Endoplasmic reticulum aminopeptidase 1 (ERAP1) is strongly associated with predilection to AS [7], has effects on HLA-B27 cell biology and cell surface expression, with ERAP1 inhibition mitigating these effects [66]. Similarly, HLA-B27 itself may also be a potential therapeutic target. Whilst targeting the entirety of surface HLA-B27 might render individuals at risk of viral infections, it might be possible to target the free heavy chain or homodimeric forms expressed on the cell surface with specific monoclonal antibodies such as HD6 [67].

## Conclusion

Despite recent advances, axial SpA still represents a therapeutic challenge with a significant unmet clinical need. Firstly, there is still no approved oral disease-modifying agent in axial SpA although several promising novel approaches are in the pipeline. Currently, our view is that the most promising oral agents may be the JAK inhibitors, which have shown efficacy in the clinical trial setting, although it remains to be determined which particular JAKs are most important in SpA. Secondly, there is currently a lack of therapeutic options for patients who are refractory to anti-TNF and anti-IL-17A therapies. Pre-clinical data suggests that blockade of GM-CSF may represent a new biological approach for these refractory patients and the safety and tolerability of GM-CSF blocking antibodies has already been shown in patients with other inflammatory diseases such as RA but a clinical trial in SpA is still needed.
